# BRAID: A Unifying Paradigm for the Analysis of Combined Drug Action

**DOI:** 10.1038/srep25523

**Published:** 2016-05-10

**Authors:** Nathaniel R. Twarog, Elizabeth Stewart, Courtney Vowell Hammill, Anang A. Shelat

**Affiliations:** 1 Departments of Chemical Biology and Therapeutics, St. Jude Children’s Research Hospital, Memphis, Tennessee 38105, USA; 2 Developmental Neurobiology St. Jude Children’s Research Hospital, Memphis, Tennessee 38105, USA

## Abstract

With combination therapies becoming increasingly vital to understanding and combatting disease, a reliable method for analyzing combined dose response is essential. The importance of combination studies both in basic and translational research necessitates a method that can be applied to a wide range of experimental and analytical conditions. However, despite increasing demand, no such unified method has materialized. Here we introduce the Bivariate Response to Additive Interacting Doses (BRAID) model, a response surface model that combines the simplicity and intuitiveness needed for basic interaction classifications with the versatility and depth needed to analyze a combined response in the context of pharmacological and toxicological constraints. We evaluate the model in a series of simulated combination experiments, a public combination dataset, and several experiments on Ewing’s Sarcoma. The resulting interaction classifications are more consistent than those produced by traditional index methods, and show a strong relationship between compound mechanisms and nature of interaction. Furthermore, analysis of fitted response surfaces in the context of pharmacological constraints yields a more concrete prediction of combination efficacy that better agrees with *in vivo* evaluations.

Combination therapies play an increasingly central role in the study and treatment of a wide variety of diseases, including infectious diseases such as tuberculosis^1^,^2^, malaria^3^,^4^, and HIV^5^,^6^,^7^, as well as many cancers^8^,^9^,^10^,^11^. By presenting the possibility of increased efficacy and reduced systemic toxicity, often by combining existing, clinically approved therapeutics, combination therapy represents one of the most fertile avenues of biomedical research, especially with the increased availability of high throughput screening and informatics technology. Combination studies can further be used to investigate the interaction of genetic and biomolecular pathways, enabling the discovery of new combination therapies^12^,^13^. Combination analysis therefore impacts nearly every stage of biomedical research, from the basic understanding of cellular pathways to the preclinical and clinical evaluation of combination therapies.

In the investigation of such therapies, of particular interest is the identification of synergistic combinations, which exhibit a stronger than expected combined effect, and the avoidance of antagonistic combinations, in which the presence of multiple therapeutics suppresses or inhibits their individual efficacies. Unfortunately, though interest in the analysis of combined action experiments is widespread and rapidly growing, there continues to be significant disagreement on how such analyses should be performed. One common reference model, Bliss independence^14^, is unsuitable for sigmoidal dose response behaviors, producing counterintuitive results in which a constant ratio combination less potent than either drug alone can be deemed synergistic^15^. Perhaps the most popular approach, the Combination Index (CI) method^16^, along with closely related methods such as the isobologram method and Interaction Index or Sum-of-FICs method^17^, suffer from conceptual and statistical limitations, some of which have been previously reported^15^,^18^,^19^, and others which shall be discussed in greater detail herein. Most challenging is the fact that CI-based methods reduce combination analysis to a simple decision between synergy, additivity, and antagonism. They provide no explicit model of a combination’s effect, and thus cannot be used to estimate the effect of a given dose or set of doses. This limitation is particularly challenging for translational research, when the reliable prediction of compound effect under real-world constraints is more essential than the identification of underlying synergy or antagonism.

The best alternative approach to address these limitations is one which employs non-linear optimization to fit a response surface model to the effects of combined compounds^19^,^20^. Response surface methods, however, including the universal response surface approach (URSA)^20^ and more recent multiparametric models^21^,^22^, have failed to see widespread use. It has been argued that such methods are overly complex^23^, but given the broad application of non-linear fitting in the analysis of single-agent pharmacology, we feel that the lack of adoption of response surface methods is due to: (a) a dearth of accessible computational tools for analysis and visualization (by comparison, CI has been implemented in free or inexpensive software systems); and (b) methodological constraints that limit the application of response surface fitting in many circumstances. Chief among these limitations is a strict adherence to the principle of Loewe additivity^24^, which requires that both compounds in a given combination exhibit the same range of effects (e.g. 0–100%). Though this constraint can be acceptable for some ligand-binding studies, partial effects in whole cell assays are not uncommon, and the constraint becomes even more untenable when the “effect” being modeled is not a proportion at all, such as an increase in enzyme activity^25^ or a rate of cell growth or death^26^.

To address these limitations, we developed a novel response surface method, the Bivariate Response to Additive Interacting Doses (BRAID) model of combined action. Inspired by the widely used Hill or log-logistic equation for single-agent dose response^27^,^28^, the eight-parameter BRAID surface model is designed to maintain a critical balance between versatility and simplicity, allowing the user to describe and capture a wide range of possible combined dose behaviors with straightforward and intuitive parameters. The model represents a unified tool for the varied goals of combination analysis, from simple classification of interaction to fully predictive modeling of a combination’s dose response behavior. Using simulated combination experiments, we show that CI-based methods produce highly variable and unpredictable statistical reliability, and these limitations are eliminated using BRAID. We also evaluate our model on publicly available combination data^13^, demonstrating a robust replication of previously observed patterns of synergy and antagonism, as well as additional insights made possible by a more precise measure of the magnitude of synergy and antagonism. Finally, we report additional insights following the application of BRAID to published experiments involving Ewing’s sarcoma (EWS)^29^.

## Results

### The BRAID model of combined action

**Figure 1:**
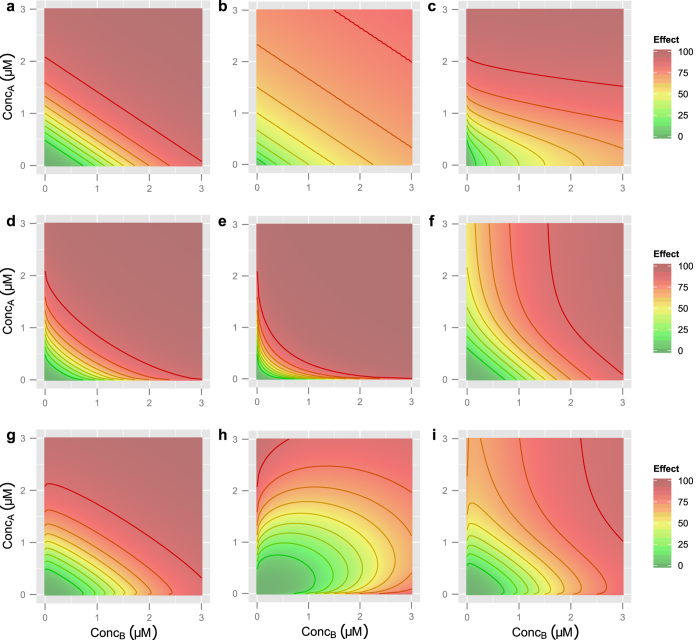
Example BRAID response surfaces. Unless otherwise specified, in all surfaces, *ID*_*M,A*_ = 1.5 μM, *ID*_*M,B*_ = 1.5 μM, *n*_*a*_ = *n*_*b*_ = 3, *E*_0_ = 0, *E*_*f,A*_ = *E*_*f,B*_ = 100, and *κ* = 0. Isoboles in all plots represent a change in effect equal to 10. (**a**) A basic additive response surface. (**b**) Lower Hill slopes (*n*_*a*_ = *n*_*b*_ = 1). (**c**) Differing Hill slopes (*n*_*a*_ = 1, *n*_*b*_ = 3). (**d**) Mild synergy (*κ* = 0.75). (**e**) Strong synergy (*κ* = 2.5). (**f**) Differing final effects (*E*_*f,A*_ = 100, *E*_*f,B*_ = 55). (**g**) Mild antagonism (*κ* = −0.3). (**h**) Severe antagonism (*κ* = −1.2). (**i**) A more complex surface, with *n*_*a*_ = 2.7, *n*_*b*_ = 3.2, *E*_*f,A*_ = 100, *E*_*f,B*_ = 75, and *κ* = −0.5. Note that the isoboles, particularly at higher effect levels, show regions of convexity and concavity; traditionally, surfaces like this would necessitate varying domains of synergy and antagonism. However, by allowing the maximal effects of the two drugs to differ, such surfaces can be modeled with a single interaction parameter.

The effect of drug combinations was fit using the BRAID model of combined action, defined in Equation 1. This eight parameter model expresses an effect as a function of two doses, using seven parameters taken from the individual dose response behavior of the two drugs in a given combination (*E*_0_, the estimated effect when neither drug is present; *E*_*f*,*A*_ and *E*_*f*,*B*_, the maximal effects of both drugs; *ID*_*M*,*A*_ and *ID*_*M*,*B*_, concentrations representing the potencies of both drugs; and *n*_*a*_ and *n*_*b*_, Hill equation parameters representing the sigmoidicity of both drugs’ dose response curves) and a single interaction parameter, *κ*, which represents the presence and magnitude of synergy or antagonism. Specifically, *κ* < 0 implies antagonism, *κ* = 0 implies additivity, and *κ* > 0 implies synergy.


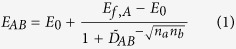



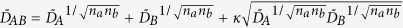



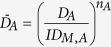


and


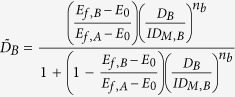


it is important to note that this equation assumes that drug *A* has an effect that is equal to or greater in magnitude than drug *B*; if this is not the case, the inputs must be reversed so that *E*_*f*,*A*_ represents the larger effect. Though algebraically daunting, the behavior of this response surface model is very straightforward, with each of the eight parameters influencing the resulting surface in a continuous, intuitive manner (Fig. 1).

Several points distinguish the BRAID model from existing response surface approaches. It is expressed in the form of an explicit eight parameter equation, meaning it can be fit using basic non-linear optimization techniques in any statistical software to any set of at least eight measurements which represent the effect of two-drug combinations in different ratios (though clearly, a larger number of data points will yield a more accurate and robust fit). It can represent the combination – with interaction – of any two dose-response behaviors represented by a Hill equation. The representation of antagonism by negative values of kappa is broader and more inclusive than many response surface models, including URSA^20^, allowing for response surfaces in which the presence of one drug reduces the potency of another. Finally, it is motivated by a fundamental property of Loewe additivity^24^: that a drug combined with itself must be additive. However, the BRAID additive surface (*κ* = 0) is not a Loewe additive surface; indeed, this is essential for its versatility, as a Loewe additive combination cannot exist between two drugs with differing maximal effects (see Supplementary Text).

### Comparison with CI using simulations

We simulated a large number of Loewe additive combination experiments to compare the statistical stability of the BRAID model to CI (see Methods). Because CI is not associated with a standardized statistic or *p*-value, designations of significant synergy or significant antagonism must be determined by largely arbitrary thresholds. Chou specifies a narrow range of CI values (0.9–1.1) as “nearly additive”, but offers no physical, statistical, or theoretical rationale for those thresholds^30^. Other journals have adopted much wider ranges for the designation of additivity^18^.

**Figure 2:**
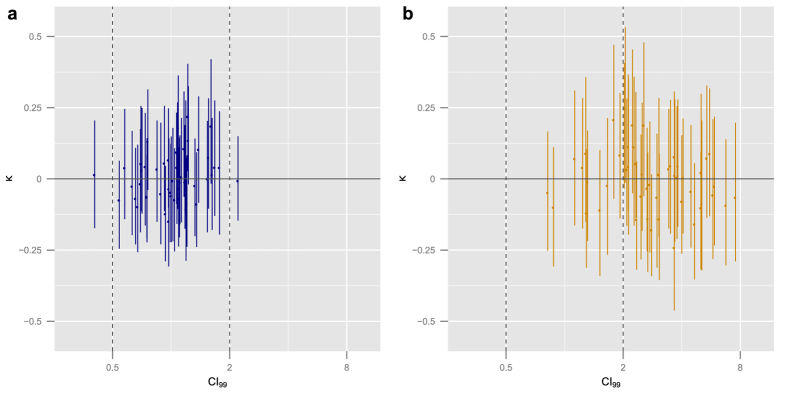
Comparison of CI and BRAID using simulations. (**a**) Estimated CI at the 99% effect level and best fit estimate of BRAID *κ* for 50 simulated additive combinations. Vertical bars indicate bootstrapped confidence intervals. In each trial, measurement noise level was 5%, and the Hill slopes of both drugs’ dose response curves were 1. Vertical dashed lines indicate the additivity range used for CI classification. (**b**) Results from simulations in which the measurement noise level was 7.5% and the Hill slope of the two drugs were 0.8 and 2.4. CI variability has substantially increased and is biased higher (due to incorrect assumptions about the shape of constant ratio combination dose response), while BRAID *κ* has remained centered around zero with a slight increase in confidence intervals.

Table 1 summarizes the results of those simulations. For comparison, we include the accuracy of the BRAID model, which lay consistently between 95% and 100% across all simulation conditions. Figure 2 graphically depicts two different sets of experimental conditions from these simulations. These experiments were designed to give CI the best chance of success, using 15 distinct concentrations for individual dose response, a 10-by-10 checkerboard for synergy analysis, and a conservative additivity range of 0.5 to 2. In spite of this, for default experimental conditions (highlighted in Table 1), CI correctly identified the combination as additive at the 99% effect level only 80% of the time. Decreasing or increasing the measurement noise in the experiment correspondingly increased or decreased CI’s accuracy, which is unsurprising. However, the fact that the statistical significance of an Index value is highly dependent on experimental precision is rarely mentioned when such values are reported in the literature.

Attempts to improve CI accuracy at the highest effect levels by shifting the range of concentrations sampled in the experiment higher were unsuccessful. Raising the center of the concentrations tested to even four-fold above the EC_50_ of the drugs being evaluated greatly reduces the accuracy of CI, both at the 99% effect level and the 50% effect level. Not only is this result counterintuitive, it also suggests that the reliability of CI at higher effect levels is dependent on knowing the EC_50_ of the drugs being tested with great precision before the combination experiment is performed. The only methodological change that improved the accuracy of CI at the 99% effect level was measuring concentrations more densely around the EC_50_, as this improved the ability of the method to estimate the Hill slopes of the two drugs.

When the Hill slopes of the two drugs differed, the performance of CI fell even further, falling below chance at the most extreme differences. This reflects not just instability, but analytical error: the CI method as described by Chou requires fitting Hill curves to constant ratio combinations^30^. When Hill slopes differ, however, constant ratio combinations in a Loewe additive response surface *do not* follow a Hill curve^15^. This misapplication of dose response modeling severely biased the estimate of CI (Fig. 2b).

One proposed solution to the designation of significant synergy or additivity in Index- and isobologram-based methods is to leverage self-vs.-self drug combinations to produce an “envelope of additivity” associated with an assay or experimental method^13^,^31^. Though this approach is a clear improvement on the arbitrary thresholds generally applied to Index methods, it still suffers from several key limitations. First, it requires the inclusion of a large number of self-vs.-self combinations – generally at least 10 or 20 – to gain an accurate model of the window of additivity. Second, it assumes that the experimental results of a self-vs.-self combination are indeed additive; though any self-vs.-self combination must by definition be additive in theory, measurement bias, dilution errors, and other forms of experimental or analytical errors can generate a response surface that is not additive in practice. Finally, it fails to account for the fact that the statistical stability of an index like CI varies not only from one experiment to the next, but from one compound to the next; so any such envelope must integrate across both highly sensitive and highly unstable experimental conditions.

To demonstrate this, we simulated a series of 1000 self-vs.-self combinations, with Hill slopes randomly selected from the set of eight Hill slopes listed in Table 1, and concentrations centered either on the EC_50_ of the tested drug or two-fold above. The resulting additivity window, modeled as a 95% confidence interval on the set of self-vs.self combination index values (at the 99% effect level), ranged from 0.34 to 2.87. This range of values is highly conservative, excluding most cases of synergy and antagonism in the literature, including those reported by Chou^30^. Yet when applied to a simulated additive combination of a drug with Hill slope 0.8 and a drug with Hill slope 2.4, it failed to identify additivity in 32.2% of trials.

### Evaluation of synergy and antagonism in a large dataset

**Figure 3:**
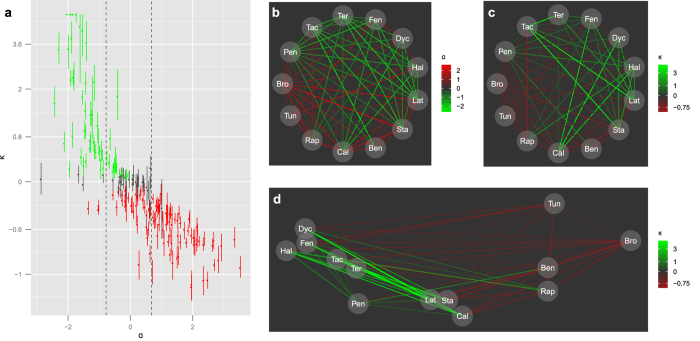
Results of BRAID analysis on a large combination dataset. (**a**) Plot of the interaction parameter *α* developed by Cokol *et al.* versus BRAID *κ* in all 200 combinations tested. Vertical bars indicate bootstrapped confidence intervals on *κ*. Points in red were designated antagonistic by BRAID analysis, while points in green were designated synergistic. Vertical dashed lines indicate the ‘window of additivity’ on *α* used by Cokol *et al.* to designate significant synergy or antagonism. (**b**) Graph of sign and magnitude of interaction between all pairs of 13 compounds, as represented by *α*. (**c**) Graph of sign and magnitude of interaction between all pairs of 13 compounds, as represented by *κ*. (**d**) Plot of all 13 compounds tested in a two-dimensional space extracted by applying multidimensional scaling to similarity of interaction as determined by *κ*.

To further evaluate our method, we applied BRAID to a publicly available combined action dataset containing 200 antifungal drug pairs reported by Cokol *et al.*^13^. Though the nature of the data was far from optimal for our analytical method, utilizing linearly spaced concentrations and a non-uniform distribution of noise in the modeled effect, it was straightforward to fit the BRAID model to each response surface. As shown in Fig. 3a, BRAID *κ* correlates well with the Cokol *et al. α* parameter (Pearson correlation = −0.82). Of the 50 combinations reported as synergistic, 43 were judged synergistic by the BRAID model; of the 72 combinations reported as antagonistic, 71 were judged antagonistic by the BRAID model.

However, BRAID analysis yielded additional insights into this dataset. Because our model integrates information from the entire measured response surface, it can better capture differences in the magnitude of synergy and antagonism. Figure 3b depicts the magnitude of *α* among 13 compounds which were evaluated in all possible pairwise combinations. Note that, though the pairs exhibit a great deal of non-additive interaction, the magnitudes of synergy and antagonism are largely flat, affording no means of further distinguishing within the sets of ‘synergizers’ and ‘antagonizers’. However, clear differences in magnitude emerge when the same set of combinations is evaluated using *κ* (Fig. 3c). For example, pentamidine, though it exhibits synergy with numerous compounds and is deemed a ‘promiscuous synergizer’, produces synergy with significantly smaller magnitude than the other promiscuous synergizers.

Using these variations in interaction magnitude, the similarity between each pair of these 13 compounds was evaluated (see Methods). Multidimensional scaling was then run on this similarity matrix to position the 13 compounds in a ‘synergy similarity space’ (Fig. 3d). The predominant dimension of this space, on the *x*-axis, is the background synergy or antagonism rate noted by Cokol *et al.* The second dimension, on the other hand, picks out differences in the magnitude and distribution of these synergies and antagonisms, highlighting biologically relevant distinctions between the six promiscuous synergizers including discrimination between the Erg1-targeting compound terbinafine (Ter) and the Erg2-targeting compounds dyclonine (Dyc), fenpropimorph (Fen) and haloperidol (Hal); and the isolation of the weak promiscuous synergizer pentamidine (Pen). When a similar analysis was run on CI values, and on the interaction parameter α derived by Cokol *et al.*, only one dimension (separating promiscuous synergizers from antagonizers) was successfully extracted; discrimination between the synergizers was not possible.

### Synergy between PARP inhibitors and DNA damaging agents

Several cancers have been shown to exhibit deficiencies in DNA double-strand break (DSB) repair that makes them particularly sensitive to inhibitors of poly (ADP-ribose) polymerase 1 (PARP1), a key component of single strand break repair in human cells^32^,^33^. Recent work has shown that EWS cell lines with the EWS-FLI translocation are sensitive to the PARP inhibitor olaparib^34^,^35^, suggesting EWS may have deficiencies in DSB repair. As EWS standard of care (SOC) agents temozolomide (TMZ) and irinotecan (IRN) act through DNA damage^36^, it is reasonable to hypothesize that these agents would interact synergistically with PARP inhibitors (PARP_i_) in EWS cell lines.

**Figure 4:**
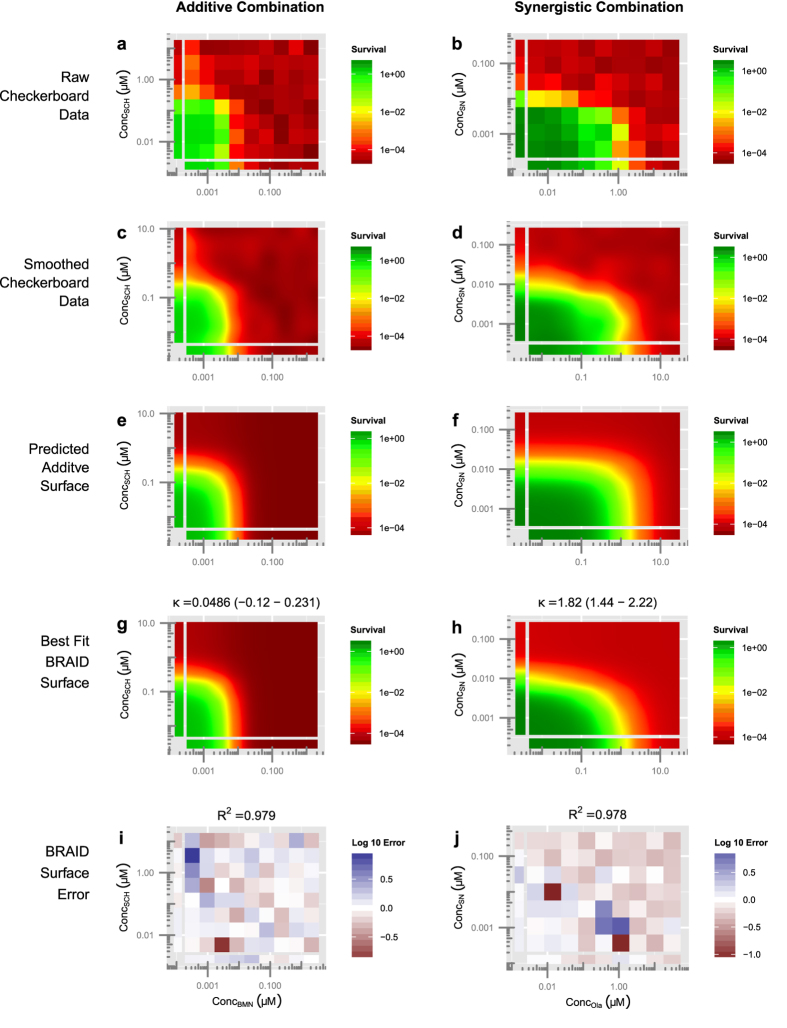
Illustration of the BRAID analysis approach. The combination of BMN-673 and SCH-900776 in cell line ES8 (**a**,**c**,**e**,**g**,**i**) and the combination of olaparib and SN-38 in cell line ES1 (**b**,**d**,**f**,**h**,**j**). (**a,b**) Cell survival (modeled by Cell-Titer Glo luminescence) is calculated relative to negative controls on all plates, and the average logarithm of each dose-pair is plotted as a response surface. (**c,d**) Smoothing data using a simple Gaussian interpolation allows the viewer to quickly evaluate the shape of the response surface. (**e,f**) The additive BRAID surface estimated using the best-fit individual dose-response parameters of the two drugs. (**g,h**) The best fit BRAID surface in which the interaction parameter *κ* is allowed to vary freely. The best fit BRAID surface for BMN-673 vs. SCH-900776 is very close to the estimated additive surface, while for olaparib vs. SN-38 it is visibly different from the additive surface. This is due to strong synergy between olaparib and SN-38. (**i,j**) Comparing the best fit BRAID surface with the measured unsmoothed data allows the viewer to quickly gauge whether the surface contains non-uniform errors, suggesting artifacts or a poor fit.

To evaluate this hypothesis, and identify effective combinations for treatment, three PARP inhibitors (BMN-673, olaparib, and veliparib) were tested in combination with TMZ and SN-38 (the active metabolite of IRN) in three EWS cell lines, (ES1, ES8, and EW8)^29^. For each combination, the BRAID equation was fit, using standard unweighted non-linear optimization, to the logarithm of cell survival, normalized to the negative controls in each assay plate (see Methods). Examples of the BRAID analysis, from the experiments in this section and the next section, are shown in Fig. 4.

The estimates of BRAID *κ*, along with bootstrapped confidence intervals, for all combinations of PARP_i_ and SOC agents are listed in Supplementary Table S1. No combination in any cell line was judged antagonistic, and only the combination of BMN-673 and irinotecan in ES8 and EW8 and the combination of veliparib and irinotecan in ES8 were judged additive; all other combinations were judged synergistic, confirming the hypothesis suggested by mechanistic considerations. Overall, combinations involving olaparib or veliparib exhibited stronger synergy than those involving BMN-673; in ES1, combinations involving irinotecan exhibited stronger synergy than those involving temozolomide, while in ES8 and EW8, the reverse was true. Based solely on degree of synergy observed across all three cell lines, the most promising combination would be olaparib and temozolomide; however, all combinations exhibit synergy in at least some cell lines.

**Figure 5:**
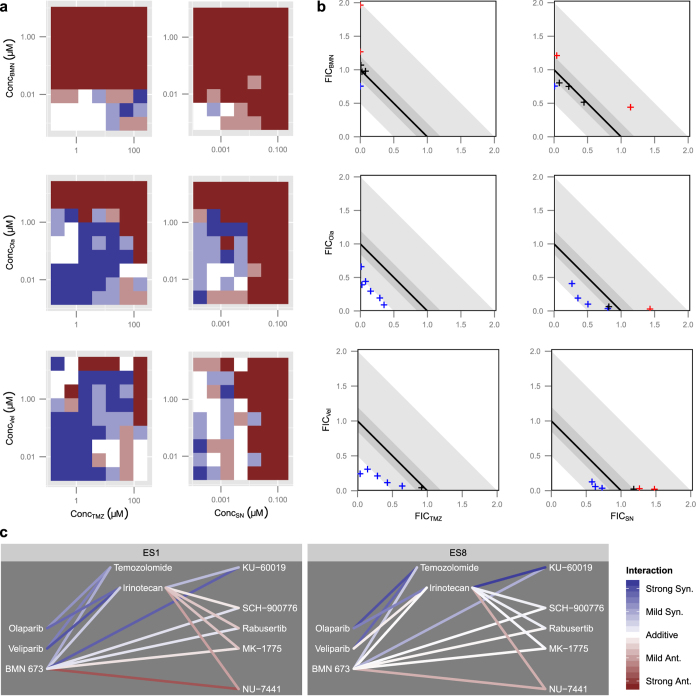
Comparison of CI-based methods and BRAID in EWS. (**a**) Plot of CI values for all dose pairs tested in the ES8 cell line. Dark blue corresponds to strong synergy (*CI* < 0.5); light blue to mild or moderate synergy (0.5 < *CI* < 0.85); white to near-additivity (0.85 < *CI* < 1.2); light red to mild or moderate antagonism (1.2 < *CI* < 2); and dark red to strong antagonism (*CI* > 2). (**b**) FIC plots for all six PARP_i_/SOC combinations in ES8 at the 99% effect level. Dark shaded grey regions indicate the areas of near-additivity, while light grey shaded regions indicate areas of mild or moderate synergy or antagonism. Points are colored according to whether they lie above, within, or below the near-additive region. (**c**) Network graphs depicting the magnitude and sign of the best fit BRAID *κ* for all tested combinations. For reference, “strong synergy” corresponds to *κ* = 2.5, “mild synergy” corresponds to *κ* = 1, “mild antagonism” corresponds to *κ* = −0.66, and “strong antagonism” corresponds to *κ* = −1.

For comparison, a more traditional analysis was run using CI^16^ and sum-of-FICs approaches^17^. The CI plots in Fig. 5a show widely varying CI values which suggest complex patterns of synergy and antagonism, but are in fact the result of the instability in CI discussed earlier. FIC plots for the 99% effect level are shown in Fig. 5b for several compound ratios in all combinations in ES8. Ratios are colored according to whether they differ from “near additivity”, and in what direction. While two combinations (olaparib vs. temozolomide and veliparib vs. temozolomide) exhibit consistent synergy, in agreement with the BRAID analysis, the remaining combinations are highly inconclusive, with some ratios exhibiting synergy, some additivity, and some antagonism. Furthermore, application of the sum-of-FICs method to combinations in ES1 and ES8 at the 90% and 99% effect levels failed to identify clear synergy between BMN-673 and either SOC agent (see Supplementary Table S2). The sum-of-FICs approach, therefore, produces a much less consistent evaluation of the nature of interaction between PARP_i_ and DNA-damaging agents than the BRAID model.

### Relationship between mechanism of action and nature of interaction

In addition to the experiments described above, six further experiments were run in which the most potent PARPi, BMN-673, and the most potent SOC agent (SN-38) were tested in ES1 and ES8 in combination with five additional compounds targeting DNA repair checkpoints: an Ataxia telangiectasia mutated (ATM) inhibitor (KU-60019), two Checkpoint kinase 1 (CHEK1) inhibitors (Rabusertib and SCH-900776), a WEE1 inhibitor (MK-1775), and a DNA-PKcs inhibitor (NU-7441). Network graphs depicting the degree and nature of interaction between these compounds are shown in Fig. 5c.

In addition to inhibiting the catalytic function of PARP enzymes, BMN-673 also generates persistent PARP1-DNA adducts that behave similarly to adducts generated by Topoisomerase I inhibitors such as irinotecan^29^. It was therefore gratifying that our method showed that BMN-673 and irinotecan exhibit similar interactions with compounds targeting DNA repair: antagonism in combination with the DNA-PK inhibitor, near additivity or mild antagonism in combination with the CHEK1 and WEE1 inhibitors, and synergy in combination with the ATM inhibitor. These results demonstrate that the robustness and versatility of the BRAID model enable us to develop a complex portrait of the impact of compound mechanism on the nature of combination interaction; furthermore, this analysis suggests that ATM inhibitors may constitute another promising set of candidates for combination therapy in EWS.

### Response surface analysis in a broader context

**Figure 6:**
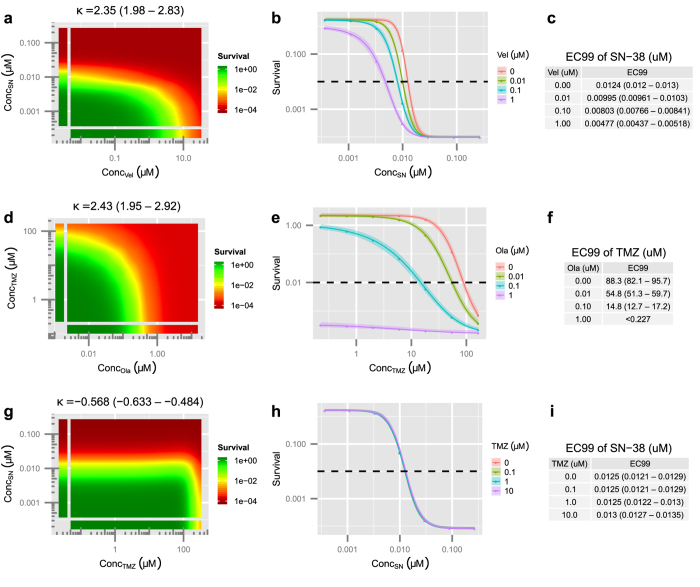
Modeling potentiation. Calculation of potentiation with the BRAID model for the combinations of veliparib and SN-38 in ES1 (**a**–**c**), olaparib and temozolomide in ES8 (**d**–**f**), and temozolomide and SN-38 in ES1 (**g**–**i**). (**a,d,g**) Best fit BRAID surface for each combination with estimated value of *κ* and confidence interval. (**b,e,h**) Estimated effect of the second drug in each combination in the presence of various levels of the first drug. Curve shifts to the left indicate potentiation. (**c,f,i**) *EC*_99_ values of the second drug in each combination in the presence of various levels of the first drug. Note that though the combinations of veliparib vs SN-38 and olaparib vs. temozolomide exhibit similar *κ* values, olaparib produces considerably more potentiation of temozolomide than veliparib does of SN-38. This is due both to the increased potency of olaparib and the lower Hill slope of temozolomide.

Though it receives more than its share of interest and attention, the presence or absence of synergy (and to a lesser extent, antagonism) is by no means the only relevant question in the evaluation of a particular drug combination, especially in translational research. A highly synergistic combination, though intriguing from a scientific standpoint, is of little use if the compounds involved cannot be delivered at sufficiently high concentrations to achieve a desired effect. Many researchers are as concerned with potentiation – the effect that a fixed amount of one compound has on the potency of another – as with synergy. The degree of potentiation in any given combination cannot be determined from the value of CI or sum-of-FICs. However, with a response surface model determined by an explicit closed-form equation, such as the BRAID model, such calculations are straightforward (see Supplementary Text and Fig. 6). Yet, even the degree of potentiation is a significant simplification which fails to take into account the behavior of a combination across a wide, clinically relevant range of doses.

**Figure 7:**
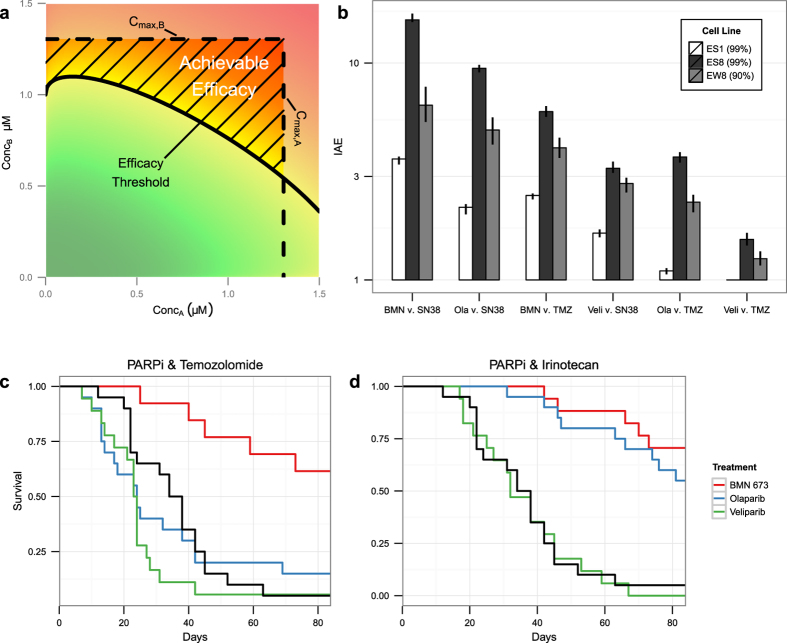
Predictions of the index of achievable efficacy (IAE). (**a**) Illustration of the calculation of IAE. The area bounded by the maximum achievable concentrations of both compounds and the isobole of the desired efficacy is the “area of achievable efficacy”. The square root of the ratio of the area of all achievable dose pairs (*C*_max,*A*_∙ *C*_max,*B*_ in this case) to the area of achievable efficacy is the IAE. (**b**) Measured values of IAE_99_ in cell lines ES1 and ES8 and IAE_90_ in cell line EW8 for all PARPi and SOC agent combinations. Black bars indicate bootstrapped confidence intervals on IAE values. (**c,d**) Survival of mice orthotopically implanted with ES8 cells treated with combinations of all three PARP_i_ with temozolomide (**c**) and irinotecan (**d**). Black survival curves indicate the SOC only treatment of irinotecan and temozolomide.

In drug discovery and translational research, the therapeutic index is commonly used as a straightforward measure of the safety and feasibility of a therapeutic candidate^37^. Though it cannot capture all of the complexities and competing factors that influence a candidate’s success *in vivo*, it provides a scale-free, experimentally-accessible means of comparing a wide range of compounds for prioritization. Using the therapeutic index as a template, we developed an evaluation measure for drug combinations designed to take advantage of the behavior of a response surface as a whole, rather than only one or two of its parameters. For each of the PARP inhibitors and SOC agents described earlier, a maximum achievable concentration was determined based on pharmacokinetic and toxicological constraints^29^. For each combination, the two maximum achievable concentrations and the best fit BRAID response surface were used to calculate the *index of achievable efficacy* (IAE) (see Supplementary Text and Fig. 7a). The IAE represents the degree to which achievable safe combinations exceed the threshold of combinations needed to achieve a desired effect. As such, it represents a much more pragmatic and concrete measure of a combination’s effectiveness than a single interaction parameter indicating synergy, additivity, or antagonism.

The set of all measured IAE_99_ values, with bootstrapped confidence intervals, is shown for all combinations of PARP_i_ with SOC agents across all EWS cell lines tested in Supplementary Table S3. The maximum efficacy in EW8 was approximately 99.7% killing, making the estimates of IAE_99_ more unstable than in ES1 and ES8; thus we have also included the measured values of IAE_90_ for that cell line. For all three EWS cell lines, we find an interesting result: the ordering of effective combinations based solely on the magnitude of synergy values has now been largely reversed (Fig. 7b). Based purely on the magnitude of the parameter *κ* across ES1, ES8, and EW8, the ordering of combinations by degree of synergy would be:


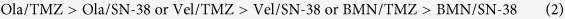


Though they achieved lower levels of synergy, the high potencies of BMN-673 and irinotecan translate to significantly higher IAE values; on the other hand, though temozolomide and veliparib often produce highly synergistic combinations, their low potencies greatly reduce the proportion of achievable dose pairs that yield a 90% or 99% effect. The ordering of combinations according to IAE is thus:


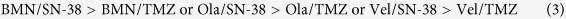


To directly evaluate the effectiveness of each PARP_i_ in combination with SOC agents, a 12-week *in vivo* efficacy study was run using mice orthotopically injected with ES8 cells^29^. The results of this study relevant to our analysis are shown in Fig. 7c,d. The most effective two-drug combination was BMN-673 and irinotecan, followed by olaparib and irinotecan and BMN-673 and temozolomide, with olaparib and temozolomide and both combinations involving veliparib having an effect comparable to the control agents. This ordering correlates well with the ordering using IAE, suggesting that this metric is a better predictor of clinical effectiveness than simple measures of synergy or antagonism. Critically, measures such as IAE can only be calculated with a model of the full response surface.

## Discussion

The R code used to produce the analyses and plots presented in this paper have been placed in two freely available R packages: *braidrm*, which contains the implementation and inversion of the BRAID equation, along with functions for fitting the surface model to given data, calculating confidence intervals, and selecting the most parsimonious model; and *braidReports*, which contains functions for plotting combined action and BRAID surfaces, calculating IAE, and producing complete BRAID analysis reports (see Supplementary Material). We include the second package, which was used to plot the response surfaces shown in this paper, as we have found a dearth of accessible and intuitive tools for visualizing combined dose response surfaces; we consider such tools to be invaluable elements of any combined action analysis toolbox.

There are additional applications of the BRAID surface model that we have not described here. By analyzing, at different time points, the degree of interaction between two competing or cooperating mechanisms, one can gain insight into the time-dependence of the interaction between mechanisms. In cases where compounds have overlapping dose-limiting toxicities, the degree of those effects could be modeled with a second BRAID surface, allowing for a more sophisticated and accurate representation of combined efficacy and safety using IAE. The BRAID equation can also be augmented with a more complex model of interaction such as that proposed by Kong and Lee^22^ which accounts for situations where the efficacy of the combination exceeds the maximum efficacy observed for each compound in isolation (see eBRAID model in the Supplementary Text).

The BRAID model provides an immediately accessible common language of combination analysis that the field clearly requires^30^,^38^, but which has to date failed to materialize. An intuitive parameter space, built on the well-established Hill dose response parameters for the individual compounds in a combination, enables the modeling and analysis of almost any two-drug combination, even those with significantly different maximal effects. As discussed in the Methods section, this feature is particularly critical when modeling the logarithmic transform of cell survival.

The use of a single interaction parameter enables the straightforward classification of synergy, additivity, or antagonism – a task until now restricted to CI-based methods – and a tool essential to the analysis of the relationship between compound mechanism and nature of interaction. When applied to a publically available dataset, our method uncovered an additional dimension of information embedded in the interaction patterns of drug combinations. When used to explore the effects of PARP_i_ and DNA-damaging agents in EWS, our method produced more consistent and robust judgments about the nature of interaction than CI and sum-of-FICs. Furthermore, the construction of valid confidence intervals, enabled by the use of an explicit parametric model rather than a calculated index, provides the researcher with a clear measure of statistical significance in such classifications.

In many research settings, however, the mere identification of synergy or antagonism is not as critical as the ability to estimate, with confidence, the behavior of a particular dose or range of doses; this is especially true in translational and preclinical settings. While we fully acknowledge that no analysis of *in vitro* experiments, no matter how sophisticated, will be able to accurately predict all the complexities that arise *in vivo*, analysis such as IAE that account for the response surface as a whole with physiologically-reasonable drug concentrations will undoubtedly provide a better metric for prioritizing compounds for further study than a simple measure of synergy or antagonism. Without an explicit model of the response surface, these and other insights are difficult to uncover; with the BRAID model, such analysis is readily accessible.

## Methods

### Data evaluation

Assay plates in each experiment were prepared using a single drugging plate for each compound; thus an error in compound concentration in a given well of the drugging plate would be replicated in each assay plate measuring the effect of that compound, either alone or in combination. To reduce the potential biasing impact of such concentration errors, we use the behavior of the compound alone to construct a Bayesian estimate of the actual compound concentration in each well, optimizing the following equation:


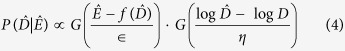


where 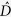 is the estimated actual dose, *Ê* is the measured effect, *D* is the intended dose, *G* is the standard normal distribution, *f* is the function describing the relationship between dose and effect, and *ϵ* and *η* are noise parameters representing imprecision in the measurement and concentration preparation. Thus we can use the measured effect and intended concentration to construct a maximum a posteriori estimate of the actual concentration in each well of each drugging plate ; this estimate can be integrated into a more robust BRAID surface fit in each combination. For the function *f*, we use the best fit Hill equation estimated from the uncorrected data. Because of the nature of the normal distribution, the right hand side of the above equation can be maximized using only the ratio *ϵ*/*η*; for base-10 logarithm of cell survival as the estimated effect and our compound preparation methods, we have found a ratio of 1.5 to produce uniform results.

### Model fitting

Data from the experiments presented in this paper were drawn from an assay measuring cell survival in which noise increases linearly with the magnitude of the assay. In non-linear optimization, such non-uniform noise is typically handled in one of two ways: using a weighted least-squares objective function, or by fitting the function in a transformed space in which the noise is uniform – in this case, a logarithmic transform. We choose the second approach (see Supplementary Text for rationale). It is worth noting that in this transformed space, the concepts of 0% and 100% effect are largely meaningless, making the fact that the BRAID model allows for differing maximal effects particularly critical.

Experimental data were fit in the R statistical computing environment using the optim non-linear optimization function^39^. The measured “effect” was the base-10 logarithm of cell survival normalized to negative controls on each assay plate. All eight parameters were bound in the optimization to reasonable ranges to prevent extreme fits; the parameters *n*_*a*_, *n*_*b*_, *ID*_*M,A*_, and *ID*_*M,B*_ were fit in a logarithmic space, while the remaining four parameters were fit linearly.

As the potency of some drugs can be highly unpredictable, in some cases the measured data are insufficient to robustly fit all of the initial and maximal effect parameters *E*_0_, *E*_*f,A*_, and *E*_*f,B*_. To prevent unstable fits in such circumstances, each combination was fit to 10 different models in which various combinations of *E*_0_, *E*_*f,A*_, and *E*_*f,B*_ were fixed to default values (defined by positive and negative controls), allowed to vary freely, or constrained to be equal to one another. The most parsimonious model was selected using the Akaike information criterion^40^ calculated for each model.

Given the most parsimonious model, 95% confidence intervals were estimated from a set of 200 bootstrapped coefficients calculated by resampling residuals and refitting the corresponding BRAID model. Once calculated, these bootstrapped coefficients could also be used to calculate 95% confidence intervals on any numerical property of the BRAID surface which varies continuously as a function of the surface parameters, including the estimated effect at a particular dose pair, the potentiation of one compound by the other, and values of the index of achievable efficacy, or IAE.

### Simulated combination experiments

For comparison of the BRAID model and CI, a series of simple combination analysis experiments was simulated in the R statistical computing environment^39^. In each simulated trial, the dose response behavior of the two virtual drugs *A* and *B* was measured at 15 concentrations in a serial dilution centered on a particular multiple of the *ID*_*M*_ of the corresponding drug. The effect of the combination was evaluated across a 10-by-10 checkerboard of dose pairs, with the 10 concentrations of each drug also centered on the same multiple of the *ID*_*M*_ of the corresponding drug. The effect of the drugs always ranged from 0% at low concentrations to 100% at high concentrations. All measurements were taken in triplicate. The measured response varied around the true response in a uniform normal distribution; in addition, the actual concentrations used to simulate the response varied log-normally around the desired concentrations, to simulate variance from imperfect control of compound concentrations.

For the CI approach, once all measurements were simulated, the dose response parameters of both individual drugs were fit using standard non-linear optimization. It was given that the minimal and maximal effects were 0% and 100%, so only the potency (*ID*_*M*_) and Hill slope of both drugs needed to be estimated. Then, the seven largest diagonals of the synergy checkerboard – representing seven different constant ratio combinations of drugs *A* and *B* – were fit similarly, and the doses of both individual drugs and all seven constant ratio combinations required to achieve 50%, 90%, and 99% effect was calculated. These values were used to estimate CI values at the 50%, 90%, and 99% effect level for the trial. For each set of drug parameters and experimental conditions, the CI was evaluated 1000 times.

For the BRAID method, the measurements for drug *A* alone, for drug *B* alone, and for the full checkerboard response surface were fit to the most parsimonious BRAID surface using the model selection approach described in the previous section. As concentrations did not inhabit corresponding positions on a drugging plate, concentration correction could not be applied. If the 95% confidence interval on the interaction parameter *κ* contained 0, the combination was deemed additive. For each set of drug parameters and experimental conditions, the BRAID method was evaluated 100 times.

For all trials, the dose of median effect of both drugs was simulated as 1 μM. Unless otherwise specified, the Hill slope of both drugs was 1, measurements were centered on the doses of median effect of both drugs (rather than 2 or more times higher), concentrations tested were separated by a 2-fold serial dilution, and measured responses varied around true responses with a standard deviation of 7.5%. Actual concentrations varied log-normally around intended concentrations with a standard deviation of ln(1.1).

### Analysis of Cokol *et al.* combination dataset

Each of the 200 response surfaces in the Cokol *et al.* dataset was analyzed using the surface fitting approach described above. Combinations were designated synergistic, antagonistic, or additive according to whether the confidence interval on the interaction parameter *κ* lay entirely above 0, lay entirely below 0, or contained 0.

Similarity space analysis was performed on the 13 compounds described by Cokol *et al.*, among which all 78 possible pairwise combinations were tested^13^. All *κ* values were transformed into a more symmetrical space by adding 2, dividing by 2, and taking the logarithm; this produced a transformed version of *κ* centered on 0 (additivity) but extending indefinitely in both directions. For each pair of compounds, the degree of interaction between each of the two compounds and the remaining 11 compounds was treated as an 11-dimensional vector; the similarity between the pair was calculated using the cosine similarity measure between these two vectors (hence, compounds showing very little interaction would show only middling similarity). The measure of similarity was linearly scaled to lie between 0 and 1, and then subtracted from 1 to give a dissimilarity measure. This dissimilarity measure was then analyzed using the cmdscale function in the R Statistical Computing Environment^39^. The two dimensions shown were the only two dimensions that were extracted.

### CI and sum-of-FICs analysis of EWS Data

To estimate the fractional inhibitory concentrations (FICs)^17^ for each combination, the Hill equation was fit to each compound alone and each constant ratio combination for which at least 5 concentrations were measured. Because FIC analysis, being based on Loewe additivity, requires that the initial and maximal effect of both drugs be equal, the initial and maximal effect of each combination was first estimated by determining the best fit BRAID surface for the combination in which *E*_*f*,*A*_ is constrained to be equal to *E*_*f*,*B*_. The Hill equation was then fit to each dose-response curve using non-linear optimization, with the potency and sigmoidicity varying and the initial and maximal effects held constant. The dose response parameters of the individual drugs were used to calculate CI for all measured dose pairs. *EC*_90_ and *EC*_99_ values were determined for each constant-ratio combination, and compared with the corresponding values for compounds in isolation to determine the FIC values.

## Additional Information

**How to cite this article**: Twarog, N. R. *et al.* BRAID: A Unifying Paradigm for the Analysis of Combined Drug Action. *Sci. Rep.* **6**, 25523; doi: 10.1038/srep25523 (2016).

## Supplementary Material

Supplementary Information

## Figures and Tables

**Table 1:** Comparing BRAID to CI using simulated combination experiments.

Measurement Noise Level					
Measurement Noise		Accuracy at 50% Effect Level	Accuracy at 90% Effect Level	Accuracy at 99% Effect Level	BRAID Accuracy
5%		1.000	0.999	0.940	1.000
7.5%		1.000	0.988	0.804	0.990
10%		1.000	0.939	0.663	0.980
15%		0.992	0.799	0.487	0.960
**Concentration Centering**					
**Fold Above EC50**		**Accuracy at 50% Effect Level**	**Accuracy at 90% Effect Level**	**Accuracy at 99% Effect Level**	**BRAID Accuracy**
1		1.000	0.988	0.804	0.990
2		1.000	0.981	0.756	1.000
4		0.735	0.979	0.549	0.970
8		0.429	0.978	0.296	0.980
16		0.071	0.910	0.101	0.960
**Concentration Sampling**					
**Serial Fold Dilution**		**Accuracy at 50% Effect Level**	**Accuracy at 90% Effect Level**	**Accuracy at 99% Effect Level**	**BRAID Accuracy**
1.25		1.000	0.991	0.722	0.990
1.5		1.000	0.994	0.824	1.000
2		1.000	0.988	0.804	0.990
2.5		1.000	0.977	0.750	0.960
3		1.000	0.963	0.720	0.960
**Differing Hill Slopes**					
****na**	****nb**	**Accuracy at 50% Effect Level**	**Accuracy at 90% Effect Level**	**Accuracy at 99% Effect Level**	**BRAID Accuracy**
1	1	1.000	0.988	0.804	0.990
1	1.5	1.000	0.995	0.817	0.980
0.9	1.8	1.000	0.994	0.690	0.950
0.8	2.4	1.000	0.990	0.421	0.970
0.75	3	1.000	0.983	0.267	0.970

‘Accuracy at 50% Effect Level’ represents the proportion of constant ratio combinations across all simulated trials in which the CI at the 50% effect level lay between 0.5 and 2. ‘Accuracy at 90% Effect Level’ and ‘Accuracy at 99% Effect Level’ were calculated similarly. ‘BRAID Accuracy’ represents the proportion of trials in which the 95% confidence interval on the interaction parameter *κ* contained 0. All simulated experiments in the first three blocks of conditions involved compounds with identical Hill parameters, and can thus be considered simulated self-vs.-self combinations.
