# Erratum: BRAID: A Unifying Paradigm for the Analysis of Combined Drug Action

**DOI:** 10.1038/srep46970

**Published:** 2018-05-17

**Authors:** Nathaniel R. Twarog, Elizabeth Stewart, Courtney Vowell Hammill, Anang A. Shelat

Scientific Reports 6: Article number: 2552310.1038/srep25523; published online: 05 10 2016; updated: 05 17 2018

The original version of this Article contained an error in the indexing of the author Anang A. Shelat. This error has now been corrected.

