# Supplementary material for: BRAID: A Unifying Paradigm for the Analysis of Combined Drug Action
Source: Sci Rep. 2016 May 10;6:25523. doi: 10.1038/srep25523 (PMC4861905; doi:10.1038/srep25523)
Supplement: Supplementary Information [file srep25523-s1.pdf]

BRAID: A Unifying Paradigm for the Analysis of Combined Drug Action

Nathaniel R. Twarog, Elizabeth Stewart, Courtney Vowell Hammill & Anang A. Shelat*

Supplementary Information

*Corresponding author

Email: anang.shelat@stjude.org (AS)

	TMZ	SN-38
ES1		
BMN-673	0.895 (0.677 – 1.164)*	0.514 (0.322 – 0.719)*
Olaparib	1.27 (0.96 – 1.58)*	1.99 (1.59 – 2.46)*
Veliparib	1.58 (1.24 – 1.98)*	2.35 (1.98 – 2.83)*
ES8		
BMN-673	0.530 (0.223 – 0.866)*	–0.036 (–0.180 – 0.157)
Olaparib	2.43 (1.95 – 2.92)*	1.54 (1.25 – 1.88)*
Veliparib	2.04 (1.56 – 2.64)*	–0.137 (–0.376 – 0.150)
EW8		
BMN-673	0.920 (0.355 – 1.644)*	0.075 (–0.515 – 0.851)
Olaparib	2.12 (1.27 – 2.97)*	1.55 (0.77 – 2.60)*
Veliparib	1.454 (0.953 – 2.089)*	0.469 (0.052 – 0.868)*

Supplementary Table S1 | Best fit values of BRAID *κ* for all combinations of PARP$_i$ and SOC agents in three EWS cell lines. Lower and upper bounds of bootstrapped confidence intervals are shown in parentheses. An asterisk (*) indicates that the parameter *κ* was fit above zero with a confidence interval not containing zero, indicating statistically significant synergy with a *p*-value less than 0.05.

ES1 (90% Effect)						
EC90 of BMN 673 (nM)		6.051 (4.703–7.464)				
EC90 of Olaparib (μM)		0.85 (0.65–1.132)				
EC90 of Veliparib (μM)		3.896 (3.295–5.734)				
EC90 of Temozolomide (μM)		321.684 (321.684–335.361)				
EC90 of Irinotecan (nM)		9.726 (8.682–11.879)				
Drug A	Drug B	Ratio	FIC_A	FIC_B	Σ FICs	Interaction
BMN 673	Temozolomide	1:2131	1.442	0.058	1.500	Antagonism
BMN 673	Temozolomide	1:710	1.459	0.019	1.478	Antagonism
BMN 673	Temozolomide	1:237	1.467	0.007	1.473	Antagonism
BMN 673	Temozolomide	1:79	1.221	0.002	1.223	Antagonism
BMN 673	Temozolomide	1:26	0.569	0.000	0.569	Synergy
BMN 673	Temozolomide	1:9	5.590	0.001	5.591	Antagonism
BMN 673	Irinotecan	1:2	0.706	0.766	1.471	Antagonism
BMN 673	Irinotecan	2:1	0.925	0.334	1.259	Antagonism
BMN 673	Irinotecan	5:1	0.957	0.115	1.072	Additivity
BMN 673	Irinotecan	15:1	0.860	0.035	0.894	Additivity
BMN 673	Irinotecan	46:1	0.327	0.004	0.332	Synergy
BMN 673	Irinotecan	139:1	0.584	0.003	0.587	Synergy
Olaparib	Temozolomide	1:871	0.175	0.403	0.578	Synergy
Olaparib	Temozolomide	1:290	0.347	0.266	0.613	Synergy
Olaparib	Temozolomide	1:97	0.392	0.100	0.492	Synergy
Olaparib	Temozolomide	1:32	0.603	0.051	0.655	Synergy
Olaparib	Temozolomide	1:11	1.090	0.031	1.121	Additivity
Olaparib	Temozolomide	1:4	0.842	0.008	0.850	Additivity
Olaparib	Irinotecan	1:1	0.014	0.888	0.902	Additivity

Olaparib	Irinotecan	4:1	0.041	0.856	0.897	Additivity
Olaparib	Irinotecan	13:1	0.074	0.509	0.582	Synergy
Olaparib	Irinotecan	38:1	0.153	0.353	0.506	Synergy
Olaparib	Irinotecan	114:1	0.414	0.318	0.733	Synergy
Olaparib	Irinotecan	341:1	0.680	0.174	0.855	Additivity
Veliparib	Temozolomide	1:804	0.082	0.794	0.876	Additivity
Veliparib	Temozolomide	1:268	0.116	0.377	0.493	Synergy
Veliparib	Temozolomide	1:89	0.275	0.298	0.573	Synergy
Veliparib	Temozolomide	1:30	0.485	0.175	0.659	Synergy
Veliparib	Temozolomide	1:10	0.845	0.102	0.946	Additivity
Veliparib	Temozolomide	1:3	1.036	0.042	1.077	Additivity
Veliparib	Irinotecan	2:1	0.004	0.971	0.975	Additivity
Veliparib	Irinotecan	5:1	0.010	0.864	0.874	Additivity
Veliparib	Irinotecan	14:1	0.020	0.593	0.613	Synergy
Veliparib	Irinotecan	41:1	0.051	0.500	0.551	Synergy
Veliparib	Irinotecan	123:1	0.116	0.377	0.493	Synergy
Veliparib	Irinotecan	369:1	0.277	0.300	0.577	Synergy
ES1 (99% Effect)						
EC99 of BMN 673 (nM)		9.648 (8.306–10.984)				
EC99 of Olaparib (µM)		1.488 (1.32–1.745)				
EC99 of Veliparib (µM)		7.936 (7.313–10.082)				
EC99 of Temozolomide (µM)		358.465 (355.52–373.228)				
EC99 of Irinotecan (nM)		13.967 (13.523–16.178)				
Drug A	Drug B	Ratio	FIC_A	FIC_B	Σ FICs	Interaction
BMN 673	Temozolomide	1:2131	1.097	0.063	1.160	Additivity
BMN 673	Temozolomide	1:710	1.281	0.024	1.306	Antagonism
BMN 673	Temozolomide	1:237	1.323	0.008	1.331	Antagonism
BMN 673	Temozolomide	1:79	1.304	0.003	1.306	Antagonism
BMN 673	Temozolomide	1:26	0.743	0.001	0.743	Synergy
BMN 673	Temozolomide	1:9	3.907	0.001	3.908	Antagonism
BMN 673	Irinotecan	1:2	0.493	0.594	1.087	Additivity
BMN 673	Irinotecan	2:1	0.745	0.299	1.045	Additivity
BMN 673	Irinotecan	5:1	0.914	0.122	1.036	Additivity
BMN 673	Irinotecan	15:1	0.794	0.035	0.829	Synergy
BMN 673	Irinotecan	46:1	0.513	0.008	0.521	Synergy
BMN 673	Irinotecan	139:1	0.572	0.003	0.574	Synergy
Olaparib	Temozolomide	1:871	0.129	0.468	0.597	Synergy
Olaparib	Temozolomide	1:290	0.266	0.320	0.586	Synergy
Olaparib	Temozolomide	1:97	0.377	0.152	0.529	Synergy
Olaparib	Temozolomide	1:32	0.596	0.080	0.676	Synergy
Olaparib	Temozolomide	1:11	0.879	0.039	0.918	Additivity
Olaparib	Temozolomide	1:4	0.742	0.011	0.753	Synergy
Olaparib	Irinotecan	1:1	0.009	0.689	0.698	Synergy
Olaparib	Irinotecan	4:1	0.026	0.664	0.690	Synergy
Olaparib	Irinotecan	13:1	0.052	0.438	0.490	Synergy
Olaparib	Irinotecan	38:1	0.120	0.336	0.456	Synergy
Olaparib	Irinotecan	114:1	0.285	0.267	0.552	Synergy
Olaparib	Irinotecan	341:1	0.526	0.164	0.690	Synergy
Veliparib	Temozolomide	1:804	0.050	0.888	0.938	Additivity
Veliparib	Temozolomide	1:268	0.077	0.459	0.536	Synergy

Veliparib	Temozolomide	1:89	0.189	0.373	0.562	Synergy
Veliparib	Temozolomide	1:30	0.375	0.247	0.623	Synergy
Veliparib	Temozolomide	1:10	0.630	0.139	0.769	Synergy
Veliparib	Temozolomide	1:3	0.845	0.062	0.907	Additivity
Veliparib	Irinotecan	2:1	0.002	0.753	0.755	Synergy
Veliparib	Irinotecan	5:1	0.005	0.671	0.676	Synergy
Veliparib	Irinotecan	14:1	0.012	0.518	0.530	Synergy
Veliparib	Irinotecan	41:1	0.032	0.443	0.475	Synergy
Veliparib	Irinotecan	123:1	0.071	0.330	0.401	Synergy
Veliparib	Irinotecan	369:1	0.151	0.233	0.384	Synergy
ES8 (90% Effect)						
EC90 of BMN 673 (nM)		1.678 (1.377–2.221)				
EC90 of Olaparib (µM)		0.206 (0.162–0.286)				
EC90 of Veliparib (µM)		1.775 (1.339–2.644)				
EC90 of Temozolomide (µM)		41.396 (29.57–49.222)				
EC90 of Irinotecan (nM)		1.018 (0.781–1.726)				
Drug A	Drug B	Ratio	FIC_A	FIC_B	Σ FICs	Interaction
BMN 673	Temozolomide	1:2131	0.898	0.078	0.976	Additivity
BMN 673	Temozolomide	1:710	0.942	0.027	0.969	Additivity
BMN 673	Temozolomide	1:237	0.992	0.010	1.001	Additivity
BMN 673	Temozolomide	1:79	0.511	0.002	0.512	Synergy
BMN 673	Temozolomide	1:26	1.974	0.002	1.976	Antagonism
BMN 673	Temozolomide	1:9	0.782	0.000	0.782	Synergy
BMN 673	Irinotecan	1:2	0.438	1.258	1.696	Antagonism
BMN 673	Irinotecan	2:1	0.480	0.459	0.939	Additivity
BMN 673	Irinotecan	5:1	0.790	0.252	1.042	Additivity
BMN 673	Irinotecan	15:1	0.602	0.064	0.666	Synergy
BMN 673	Irinotecan	46:1	0.814	0.029	0.842	Synergy
BMN 673	Irinotecan	139:1	0.334	0.004	0.338	Synergy
Olaparib	Temozolomide	1:871	0.092	0.398	0.490	Synergy
Olaparib	Temozolomide	1:290	0.187	0.270	0.457	Synergy
Olaparib	Temozolomide	1:97	0.311	0.150	0.461	Synergy
Olaparib	Temozolomide	1:32	0.371	0.060	0.430	Synergy
Olaparib	Temozolomide	1:11	0.508	0.027	0.536	Synergy
Olaparib	Temozolomide	1:4	0.966	0.017	0.983	Additivity
Olaparib	Irinotecan	1:1	0.011	1.654	1.665	Antagonism
Olaparib	Irinotecan	4:1	0.019	0.924	0.943	Additivity
Olaparib	Irinotecan	13:1	0.050	0.807	0.857	Additivity
Olaparib	Irinotecan	38:1	0.118	0.632	0.750	Synergy
Olaparib	Irinotecan	114:1	0.234	0.416	0.650	Synergy
Olaparib	Irinotecan	341:1	0.414	0.246	0.660	Synergy
Veliparib	Temozolomide	1:804	0.037	1.291	1.328	Antagonism
Veliparib	Temozolomide	1:268	0.060	0.693	0.754	Synergy
Veliparib	Temozolomide	1:89	0.124	0.475	0.599	Synergy
Veliparib	Temozolomide	1:30	0.204	0.261	0.465	Synergy
Veliparib	Temozolomide	1:10	0.414	0.176	0.590	Synergy
Veliparib	Temozolomide	1:3	0.178	0.025	0.203	Synergy
Veliparib	Irinotecan	2:1	0.001	1.698	1.699	Antagonism
Veliparib	Irinotecan	5:1	0.003	1.128	1.131	Additivity
Veliparib	Irinotecan	14:1	0.010	1.283	1.293	Antagonism

Veliparib	Irinotecan	41:1	0.021	0.884	0.905	Additivity
Veliparib	Irinotecan	123:1	0.051	0.727	0.778	Synergy
Veliparib	Irinotecan	369:1	0.142	0.672	0.814	Synergy
ES8 (99% Effect)						
EC99 of BMN 673 (nM)		2.467 (2.259–2.766)				
EC99 of Olaparib (μM)		0.374 (0.341–0.453)				
EC99 of Veliparib (μM)		3.231 (2.905–4.142)				
EC99 of Temozolomide (μM)		64.859 (54.331–68.191)				
EC99 of Irinotecan (nM)		1.586 (1.416–1.918)				
Drug A	Drug B	Ratio	FIC_A	FIC_B	Σ FICs	Interaction
BMN 673	Temozolomide	1:2131	0.959	0.078	1.037	Additivity
BMN 673	Temozolomide	1:710	0.950	0.026	0.976	Additivity
BMN 673	Temozolomide	1:237	1.049	0.009	1.058	Additivity
BMN 673	Temozolomide	1:79	0.735	0.002	0.737	Synergy
BMN 673	Temozolomide	1:26	1.944	0.002	1.946	Antagonism
BMN 673	Temozolomide	1:9	1.247	0.000	1.247	Antagonism
BMN 673	Irinotecan	1:2	0.421	1.141	1.562	Antagonism
BMN 673	Irinotecan	2:1	0.494	0.447	0.941	Additivity
BMN 673	Irinotecan	5:1	0.731	0.220	0.951	Additivity
BMN 673	Irinotecan	15:1	0.783	0.079	0.862	Additivity
BMN 673	Irinotecan	46:1	1.192	0.040	1.231	Antagonism
BMN 673	Irinotecan	139:1	0.736	0.008	0.744	Synergy
Olaparib	Temozolomide	1:871	0.070	0.353	0.424	Synergy
Olaparib	Temozolomide	1:290	0.174	0.291	0.465	Synergy
Olaparib	Temozolomide	1:97	0.276	0.154	0.429	Synergy
Olaparib	Temozolomide	1:32	0.421	0.078	0.499	Synergy
Olaparib	Temozolomide	1:11	0.371	0.023	0.394	Synergy
Olaparib	Temozolomide	1:4	0.640	0.013	0.653	Synergy
Olaparib	Irinotecan	1:1	0.009	1.432	1.440	Antagonism
Olaparib	Irinotecan	4:1	0.014	0.802	0.817	Synergy
Olaparib	Irinotecan	13:1	0.044	0.817	0.861	Additivity
Olaparib	Irinotecan	38:1	0.081	0.506	0.587	Synergy
Olaparib	Irinotecan	114:1	0.172	0.356	0.528	Synergy
Olaparib	Irinotecan	341:1	0.388	0.268	0.655	Synergy
Veliparib	Temozolomide	1:804	0.023	0.918	0.941	Additivity
Veliparib	Temozolomide	1:268	0.048	0.640	0.688	Synergy
Veliparib	Temozolomide	1:89	0.096	0.428	0.524	Synergy
Veliparib	Temozolomide	1:30	0.192	0.285	0.477	Synergy
Veliparib	Temozolomide	1:10	0.289	0.143	0.432	Synergy
Veliparib	Temozolomide	1:3	0.223	0.037	0.260	Synergy
Veliparib	Irinotecan	2:1	0.001	1.479	1.480	Antagonism
Veliparib	Irinotecan	5:1	0.003	1.182	1.185	Additivity
Veliparib	Irinotecan	14:1	0.008	1.264	1.273	Antagonism
Veliparib	Irinotecan	41:1	0.015	0.728	0.743	Synergy
Veliparib	Irinotecan	123:1	0.038	0.631	0.669	Synergy
Veliparib	Irinotecan	369:1	0.106	0.584	0.689	Synergy

Supplementary Table S2 | Fractional inhibitory concentration (FIC) values for all six PARP$_i$/SOC combinations in both ES1 and ES8 cell lines at the 90% and 99% effect level. A ratio combination is classified as synergistic if the sum of FICs is below 0.85 (see Figure 5); it is classified as antagonistic if the FIC pair lies above 1.2.

	TMZ	SN-38
ES1		
BMN-673	2.451 (2.351 – 2.504)	3.611 (3.404 – 3.704)
Olaparib	1.101 (1.062 – 1.133)	2.162 (2.006 – 2.237)
Veliparib	1 (1 – 1)	1.642 (1.572 – 1.709)
ES8		
BMN-673	5.966 (5.643 – 6.325)	15.811 (15.430 – 16.903)
Olaparib	3.689 (3.477 – 3.884)	9.449 (9.054 – 9.806)
Veliparib	1.538 (1.442 – 1.648)	3.272 (3.176 – 3.509)
EW8		
BMN-673	1.328 (1.149 – 1.538)	1 (1 – 1.014)
Olaparib	1.033 (1.000 – 1.077)	1.122 (1.028 – 1.281)
Veliparib	1 (1 – 1)	1.509 (1.270 – 1.646)
EW8 (IAE_{90})		
BMN-673	4.066 (3.651 – 4.522)	6.389 (5.345 – 7.762)
Olaparib	2.284 (2.052 – 2.469)	4.915 (4.211 – 5.617)
Veliparib	1.253 (1.166 – 1.354)	2.782 (2.532 – 2.957)

Supplementary Table S3 | Index of achievable efficacy (IAE) values for 99% killing for all six PARP$_i$/SOC combinations in three EWS cell lines (and IAE_{90} values for EW8). Numbers in parentheses indicate bootstrapped 95% confidence intervals.

SUPPLEMENTARY TEXT

Derivation of the BRAID Model

To describe the combined effect of two doses of compounds A and B, the BRAID model assumes that the individual effects of both compounds can be modeled by a Hill or log-logistic equation[1]:

$$E_A(D_A) = E_0 + \frac{E_{f,A} - E_0}{1 + \left(\frac{D_A}{ID_{M,A}}\right)^{-n_a}}$$

$$E_B(D_B) = E_0 + \frac{E_{f,B} - E_0}{1 + \left(\frac{D_B}{ID_{M,B}}\right)^{-n_b}}$$

Here, E_0 represents the predicted effect when neither drug is present, $E_{f,A}$ and $E_{f,B}$ represent the maximal effect of compounds A and B, $ID_{M,A}$ and $ID_{M,B}$ represent the doses of median effect of compounds A and B (that is, the doses of A and B alone that yield an effect halfway between E_0 and the corresponding maximal effect), and n_a and n_b are the Hill slopes or sigmoidicities of compounds A and B.

A Loewe additive combination is one that everywhere satisfies the equation

$$1 = \frac{D_A}{ID_{X,A}} + \frac{D_B}{ID_{X,B}}$$

where X is the combined effect of D_A and D_B, and $ID_{X,A}$ and $ID_{X,B}$ are the concentrations of drug A and B alone required to produce the effect X. Thus, any effect produced by any combination of drugs A and B must be also produced at some concentration by both drugs A and B in isolation; one consequence of this is that both drugs must produce the same range of effects, and have the same maximal effects. We begin with the equation for a Loewe additive surface when Hill slopes and maximal effects are equal:

$$E_{AB}(D_A, D_B) = E_0 + \frac{E_f - E_0}{1 + \left(\frac{D_A}{ID_{M,A}} + \frac{D_B}{ID_{M,B}}\right)^{-n}}$$

Though this equation does not allow for differing maximal effects or Hill slopes, we suppose that the overall form of the equation is appropriate, and that a more general equation can be written:

$$E_{AB}(D_A, D_B) = E_0 + \frac{E_f - E_0}{1 + \left(f_A(D_A) + f_B(D_B)\right)^{-n}}$$

Setting, D_A or D_B equal to zero and solving for $E_A(D_A)$ and $E_B(D_B)$ yields:

$$f_A(D_A) = \left(\frac{\left(\frac{E_{f,A} - E_0}{E_f - E_0}\right)\left(\frac{D_A}{ID_{M,A}}\right)^{n_a}}{1 + \left(1 - \frac{E_{f,A} - E_0}{E_f - E_0}\right)\left(\frac{D_B}{ID_{M,A}}\right)^{n_a}}\right)^{1/n}$$

$$f_B(D_B) = \left(\frac{\left(\frac{E_{f,B} - E_0}{E_f - E_0}\right)\left(\frac{D_B}{ID_{M,B}}\right)^{n_b}}{1 + \left(1 - \frac{E_{f,B} - E_0}{E_f - E_0}\right)\left(\frac{D_B}{ID_{M,B}}\right)^{n_b}}\right)^{1/n}$$

In order for this equation to give an additive surface when $E_f = E_{f,A} = E_{f,B}$ and $n_a = n_b$, it must be that $n = n_a = n_b$. Thus, more generally, n should be a symmetric function of n_a and n_b that simplifies to either value when they are equal. Given the multiplicative nature of exponents, the geometric mean seems most appropriate.

If we assume (without loss of arbitrariness) that $E_{f,A}$ is the larger effect of $E_{f,A}$ and $E_{f,B}$, and that $E_f = E_{f,A}$, then we get the following equation, the BRAID model of additive combined action:

$$E_{AB}(D_A, D_B) = E_0 + \frac{E_{f,A} - E_0}{1 + \left[\widetilde{D}_A^{\frac{1}{\sqrt{n_a n_b}}} + \widetilde{D}_B^{\frac{1}{\sqrt{n_a n_b}}}\right]^{-\sqrt{n_a n_b}}}$$

$$\widetilde{D}_A = \left(\frac{D_A}{ID_{M,A}}\right)^{n_a} \quad \text{and} \quad \widetilde{D}_B = \frac{\left(\frac{E_{f,B} - E_0}{E_{f,A} - E_0}\right)\left(\frac{D_B}{ID_{M,B}}\right)^{n_b}}{1 + \left(1 - \frac{E_{f,B} - E_0}{E_{f,A} - E_0}\right)\left(\frac{D_B}{ID_{M,B}}\right)^{n_b}}$$

Note that this equation assumes that

$$\frac{E_{f,A} - E_0}{E_{f,B} - E_0} \geq 1$$

That is, the effects of drug *A* and *B* are in the same direction, and the maximal effect of drug *A* is at least as large as that of drug *B*. It is easy to show that this equation simplifies to the behavior of each drug alone when the other drug is not present; that is, $E_{AB}(D_A,0) = E_A(D_A)$ and $E_{AB}(0,D_B) = E_B(D_B)$.

Substituting E_f and *n* back into the above equation and expressing $\widetilde{D}_A$ and $\widetilde{D}_B$ in terms of $E_A(D_A)$ and $E_B(D_B)$ allows us to write the following implicit form of the additive BRAID equation:

$$\left(\frac{E_f - E_{AB}}{E_{AB} - E_0}\right)^{\frac{1}{n}} = \left(\frac{E_f - E_A}{E_A - E_0}\right)^{\frac{1}{n}} + \left(\frac{E_f - E_B}{E_B - E_0}\right)^{\frac{1}{n}}$$

Given that this equation describes the BRAID model of additivity, a reasonable method for introducing interaction would be to add a multiplicative interaction term to the equation. However, if this interaction term involves the products of the two terms on the right hand side of the equation, than any negative coefficient on the interaction term will create areas of dose-pair-space that produce undefined effects. We therefore introduce an interaction term that is the geometric mean of the two terms on the right hand side, inspired by Greco, Park and Rustum[2]:

$$\left(\frac{E_f - E_{AB}}{E_{AB} - E_0}\right)^{\frac{1}{n}} = \left(\frac{E_f - E_A}{E_A - E_0}\right)^{\frac{1}{n}} + \left(\frac{E_f - E_B}{E_B - E_0}\right)^{\frac{1}{n}} + \kappa\left(\frac{E_f - E_A}{E_A - E_0}\right)^{\frac{1}{2n}}\left(\frac{E_f - E_B}{E_B - E_0}\right)^{\frac{1}{2n}}$$

As long as κ is constrained to be greater than -2, the right hand side of this equation is greater than or equal to 0 for all dose pairs, and the BRAID response surface is well defined. Converting this equation back to an explicit form produces the full BRAID model of combined action:

$$E_{AB}(D_A, D_B) = E_0 + \frac{E_{f,A} - E_0}{1 + \widetilde{D}_{AB}^{\;-\sqrt{n_a n_b}}}$$

$$\widetilde{D}_{AB} = \widetilde{D}_A^{\frac{1}{\sqrt{n_a n_b}}} + \widetilde{D}_B^{\frac{1}{\sqrt{n_a n_b}}} + \kappa\sqrt{\widetilde{D}_A^{\frac{1}{\sqrt{n_a n_b}}}\widetilde{D}_B^{\frac{1}{\sqrt{n_a n_b}}}}$$

$$\widetilde{D}_A = \left(\frac{D_A}{ID_{M,A}}\right)^{n_a} \quad \text{and} \quad \widetilde{D}_B = \frac{\left(\frac{E_{f,B} - E_0}{E_{f,A} - E_0}\right)\left(\frac{D_B}{ID_{M,B}}\right)^{n_b}}{1 + \left(1 - \frac{E_{f,B} - E_0}{E_{f,A} - E_0}\right)\left(\frac{D_B}{ID_{M,B}}\right)^{n_b}}$$

Interestingly, an alternative model of interaction can be achieved by altering the exponent in the denominator of the additive BRAID model, rather than the base. By adjusting the exponent $\sqrt{n_a n_b}$ with a multiplicative factor δ, one gets what we call the delta-BRAID model:

$$E_{AB}(D_A, D_B) = E_0 + \frac{E_{f,A} - E_0}{1 + \left[\widetilde{D}_A^{\frac{1}{\delta\sqrt{n_a n_b}}} + \widetilde{D}_B^{\frac{1}{\delta\sqrt{n_a n_b}}}\right]^{-\delta\sqrt{n_a n_b}}}$$

$$\widetilde{D}_A = \left(\frac{D_A}{ID_{M,A}}\right)^{n_a} \quad \text{and} \quad \widetilde{D}_B = \frac{\left(\frac{E_{f,B} - E_0}{E_{f,A} - E_0}\right)\left(\frac{D_B}{ID_{M,B}}\right)^{n_b}}{1 + \left(1 - \frac{E_{f,B} - E_0}{E_{f,A} - E_0}\right)\left(\frac{D_B}{ID_{M,B}}\right)^{n_b}}$$

In this model, $\delta > 1$ implies synergy, $\delta < 1$ implies antagonism, and $\delta = 1$ implies additivity. The synergistic surfaces produced by the delta-BRAID model are very similar to those produced by the standard BRAID model, but the antagonistic surfaces differ drastically, behaving in a similar fashion to the antagonistic surfaces produced by the model of Greco, Park, and Rustum[2]. Though this model is in some ways more elegant than the standard BRAID model, we find its results to be unsatisfactory; in particular, even extreme δ-antagonism cannot create surfaces in which the potency of one drug is reduced by the presence of another. Though some have argued that such response surfaces are invalid[2], we have observed such effects in several experiments, and have thus found the delta-BRAID model to be insufficient.

An additional complication arises if one expects the maximal effect of a combination to differ from the maximal effect of either drug alone. Though we have not observed this in practice, it is theoretically conceivable, and thus worth considering. Fortunately, it is not difficult to adjust the standard BRAID model to account for such a circumstance. To incorporate the widest range of possible response surfaces, including both kappa- and delta-interaction, and a differing combined maximal effect, we developed the 10 parameter extended-BRAID or eBRAID model:

$$E_{AB}(D_A, D_B) = E_0 + \frac{E_f - E_0}{1 + \widetilde{D}_{AB}^{\;-\delta\sqrt{n_a n_b}}}$$

$$\widetilde{D}_{AB} = \widetilde{D}_A^{\frac{1}{\delta\sqrt{n_a n_b}}} + \widetilde{D}_B^{\frac{1}{\delta\sqrt{n_a n_b}}} + \kappa\sqrt{\widetilde{D}_A^{\frac{1}{\delta\sqrt{n_a n_b}}}\widetilde{D}_B^{\frac{1}{\delta\sqrt{n_a n_b}}}}$$

$$\widetilde{D}_A = \frac{\left(\frac{E_{f,A} - E_0}{E_f - E_0}\right)\left(\frac{D_A}{ID_{M,B}}\right)^{n_a}}{1 + \left(1 - \frac{E_{f,A} - E_0}{E_f - E_0}\right)\left(\frac{D_A}{ID_{M,A}}\right)^{n_a}}$$

$$\widetilde{D}_B = \frac{\left(\frac{E_{f,B} - E_0}{E_f - E_0}\right)\left(\frac{D_B}{ID_{M,B}}\right)^{n_b}}{1 + \left(1 - \frac{E_{f,B} - E_0}{E_f - E_0}\right)\left(\frac{D_B}{ID_{M,B}}\right)^{n_b}}$$

In practice, we have found the eight parameter standard BRAID model to be entirely sufficient; nevertheless, all functions in the *braidrm* R package support the full 10-parameter eBRAID model if desired.

Derivation of Bayesian Concentration Correction

If we assume that the true underlying relationship between concentration and effect for a single compound is modeled by a function $f(\cdot)$, and that the measured value of an effect $\hat{E}$ is normally distributed around the true underlying effect E, then the probability of a measured effect $\hat{E}$ given an actual dose $\widehat{D}$ is

$$P(\hat{E}|\widehat{D}) = G\left(\frac{\hat{E} - f(\widehat{D})}{\epsilon}\right)$$

where $G(\cdot)$ is a standard normal distribution and ϵ is the standard deviation of the experimental noise. Furthermore, if we assume that the actual concentration $\widehat{D}$ underlying a measurement is log-normally distributed around the intended concentration D, then the probability of a particular actual concentration is

$$P(\widehat{D}) = G\left(\frac{\log\widehat{D} - \log D}{\eta}\right)$$

where η is the standard log-deviation of the concentration error. According to Bayes' rule,

$$P(\widehat{D}|\hat{E}) = \frac{P(\hat{E}|\widehat{D})P(\widehat{D})}{P(\hat{E})} \propto P(\hat{E}|\widehat{D})P(\widehat{D}) = G\left(\frac{\hat{E} - f(\widehat{D})}{\epsilon}\right)G\left(\frac{\log\widehat{D} - \log D}{\eta}\right)$$

Rationale for Fitting Logarithmic Transform of Cell Survival

Recent approaches to pharmacodynamic modeling have extended use of the Hill equation from a model of static equilibria of reactions to a component of dynamic models in which the Hill equation describes the rate at which a particular process or event (e.g. cell growth/killing) occurs[3–5]. For example, the growth or death of a particular cell population might be modeled as

$$\frac{dN}{dt} = \left(\lambda - \frac{\varepsilon}{1 + \left(\frac{D}{ID_M}\right)^{-n}} \right) N$$

where N is the population size at time t, λ is a baseline growth rate, and ε is a maximal kill rate for a particular drug. Following this approach, we can model the growth or death of a cell population in the presence of a drug *combination* as

$$\frac{dN}{dt} = \left(\lambda - \Sigma(D_A, D_B)\right) N$$

where $\Sigma(D_A,D_B)$ is an instance of the BRAID model with $E_0 = 0$. Because λ is a constant and $\Sigma(D_A,D_B)$ is not time-dependent, this differential equation can be solved easily, leading to the conclusion that after a particular exposure time t_e the predicted cell population size as a function of both doses will be:

$$N(D_A, D_B) = N_0 \exp\left(\lambda t_e - t_e \Sigma(D_A, D_B)\right)$$

and the logarithm of the resulting population size is:

$$\log N(D_A, D_B) = (\log N_0 + \lambda t_e) - t_e \Sigma(D_A, D_B)$$

It is easy to see that the right-hand side of this equation is another instance of the BRAID model, this time with $E_0 = \log N_0 + \lambda t_e$. Hence, fitting the BRAID model to the logarithmic transform of the measured cell population is appropriate both from a statistical and a mechanistic viewpoint.

Potentiation and IAE Calculations

To determine the potentiation of a compound A by the presence of a dose D_B of a second compound B at a particular effect level E, one must know the dose of A alone required to produce the effect E and the dose of A required to produce the effect E in combination with the dose D_B of B that produces the same effect. In the case of the BRAID surface model, this requires a partial inversion of the BRAID equation. Fortunately, this task, though cumbersome, is relatively straightforward. On the assumption that $E_0 < E < E_{f,A}$ and

$$\tilde{D}_B^{\frac{1}{\sqrt{n_a n_b}}} < \begin{cases} \left(\frac{E - E_0}{E_{f,A} - E}\right)^{\frac{1}{\sqrt{n_a n_b}}} & \kappa \geq 0 \\ \frac{4}{4-\kappa^2}\left(\frac{E - E_0}{E_{f,A} - E}\right)^{\frac{1}{\sqrt{n_a n_b}}} & \kappa < 0 \end{cases}$$

then we can determined the desired dose D_A by first solving for $\tilde{D}_A$:

$$\tilde{D}_A = \left(\left(\frac{E - E_0}{E_{f,A} - E}\right)^{\frac{1}{\sqrt{n_a n_b}}} + \left(\frac{\kappa^2}{2} - 1\right)\tilde{D}_B^{\frac{1}{\sqrt{n_a n_b}}} + |\kappa|\sqrt{\left(\frac{\kappa^2}{4} - 1\right)\tilde{D}_B^{\frac{2}{\sqrt{n_a n_b}}} + \left(\frac{E - E_0}{E_{f,A} - E}\right)^{\frac{1}{\sqrt{n_a n_b}}}\tilde{D}_B^{\frac{1}{\sqrt{n_a n_b}}}}\right)^{\sqrt{n_a n_b}}$$

$$E_{AB}{}^{-1}(E, \sim, D_B) = D_A = ID_{M,A}\left(\tilde{D}_A^{\frac{1}{n_a}}\right)$$

The process for determining a dose of compound *B* which produces an effect *E* in combination with a dose D_A of compound *A* proceeds similarly. It is worth noting that when κ is negative, some inputs produce two solutions; the equation above gives the larger, more conservative solution. All of these calculations are performed by the R function `invertBRAIDrsm` in the *braidrm* package. The potentiation can then be described by taking the ratio of the amount of compound *A* needed to produce the effect *E* alone ($E_{AB}{}^{-1}(E, \sim, 0)$) with amount needed in the presence of dose D_B ($E_{AB}{}^{-1}(E, \sim, D_B)$).

The traditional therapeutic index consists of the ratio of the maximum achievable dose subject to certain constraints (pharmacokinetics, toxicity, metabolism, etc.) to the minimum dose required to produce a desired effect[6]. This is equivalent to calculating the ratio of the range of achievable doses to the range of doses too low to produce the desired effect. Performing the analogous calculation for dose-pairs gives the index of achievable efficacy, or IAE. The IAE for a given combination at an effect level *E* is given by:

$$IAE_E = \left(\frac{\iint_{AC} dD_A dD_B}{\iint_{AC} dD_A dD_B - \iint_{AC} H\left(\frac{E_{AB}(D_A, D_B) - E}{E_{f,A} - E}\right) dD_A dD_B}\right)^{1/2}$$

where $H(\cdot)$ is the Heaviside step function equal to 1 for values greater than 0, and 0 for values less than 0, and AC is the space of achievable dose pairs given pharmacological constraints such as solubility and toxicity. The inclusion of $E_{f,A} - E$ simply ensures that the input to the step function has the appropriate sign. Taking the square root keeps the IAE at the same scale as the therapeutic index, so that, for example, the IAE of a drug combined with itself will be the same as the therapeutic index of that drug alone.

In the simplest case AC can be considered a rectangle of dose-pairs bounded by the maximum achievable concentration of either drug; this is assumed in the IAE calculations reported in our paper. The form of AC, however, may be much more complicated: if both drugs are identical, for example, AC is bounded above by a diagonal of slope –1 representing dose pairs whose concentrations sum to the maximum achievable concentration of the drug. AC may even be determined by a threshold placed on another BRAID surface representing overlapping toxicity. In its current form, the function `calculateIAE` in the *braidReports* package supports only the use of a rectangle of achievable doses if the drugs are different, or the lower triangle of achievable doses if the two drugs are the same.

In practice, it is not possible to calculate this quantity analytically; but it can be estimated to any desired level of accuracy by calculating the predicted BRAID effect at N points in the space of achievable dose pairs. We use a 300-by-300 grid of dose pairs within the bounding box of achievable dose pairs to generate estimates of the IAE and corresponding 95% confidence intervals.

REFERENCES

1. Hill, A.V. The possible effects of the aggregation of the molecules of hæmoglobin on its dissociation curves. *J. Physiol.* **40,** iv-vii (1910).
2. Greco, W.R., Park, H.S. & Rustum, Y.M. Application of a New Approach for the Quantitation of Drug Synergism to the Combination of cis-Diamminedichloroplatinum and 1-β-d-Arabinofuranosylcytosine. *Cancer Res.* **50,** 5318-5327 (1990).
3. Zhi, J., Nightingale, C.H. & Quintiliani, R. A pharmacodynamic model for the activity of antibiotics against microorganisms under nonsaturable conditions. *J. Pharm. Sci.* **75,** 1063-1067 (1986).
4. Zhi, J., Nightingale, C.H. & Quintiliani, R. Microbial pharmacodynamics of piperacillin in neutropenic mice of systematic infection due to Pseudomonas aeruginosa. *J. Pharmacokinet. Biop.* **16,** 355-375 (1988).
5. Mouton, J.W. & Vinks, A.A. Pharmacokinetic/Pharmacodynamic Modelling of Antibacterials In Vitro and In Vivo Using Bacterial Growth and Kill Kinetics. *Clin. Pharmacokinet.* **44,** 201-210 (2005).
